# Hub Long Noncoding RNAs with m6A Modification for Signatures and Prognostic Values in Kidney Renal Clear Cell Carcinoma

**DOI:** 10.3389/fmolb.2021.682471

**Published:** 2021-07-06

**Authors:** Gaoteng Lin, Huadong Wang, Yuqi Wu, Keruo Wang, Gang Li

**Affiliations:** ^1^ Department of Urology, Tianjin Institute of Urology, The Second Hospital of Tianjin Medical University, Tianjin, China; ^2^ Department of Urology, Tianjin Baodi Hospital, Baodi Clinical College of Tianjin Medical University, Tianjin, China

**Keywords:** kidney renal clear cell carcinoma, long noncoding RNA, N6-methyladenosine, prognostic index, WGCNA

## Abstract

**Background:** N6-methyladenosine (m6A)–modified long noncoding RNAs (m6A-lncRNAs) have been proven to be involving in regulating tumorigenesis, invasion, and metastasis for a variety of tumors. The present study aimed to screen lncRNAs with m6A modification and investigate their biological signatures and prognostic values in kidney renal clear cell carcinoma (KIRC).

**Materials and Methods:** lncRNA-seq, miRNA-seq, and mRNA-seq profiles of KIRC samples and the clinical characteristics of corresponding patients were downloaded from The Cancer Genome Atlas (TCGA). The R package “edgeR” was utilized to perform differentially expressed analysis on these profiles to gain DElncRNAs, DEmiRNAs, and DEmRNAs, respectively. The results of intersection of DElncRNAs and m6A-modified genes were analyzed by the weighted gene co-expression network analysis (WGCNA) to screen hub m6A-lncRNAs. Then, WGCNA was also used to construct an lncRNA-miRNA-mRNA (ceRNA) network. The Cox regression analysis was conducted on hub m6A-lncRNAs to construct the m6A-lncRNAs prognostic index (m6AlRsPI). Receiver operating characteristic (ROC) curve was used to assess the predictive ability of m6AlRsPI. The m6AlRsPI model was tested by internal and external cohorts. The molecular signatures and prognosis for hub m6A-lncRNAs and m6AlRsPI were analyzed. The expression level of hub m6A-lncRNAs in KIRC cell lines were quantified by qRT-PCR.

**Results:** A total of 21 hub m6A-lncRNAs associated with tumor metastasis were identified in the light of WGCNA. The ceRNA network for 21 hub m6A-lncRNAs was developed. The Cox regression analysis was performed on the 21 hub m6A-lncRNAs, screening two m6A-lncRNAs regarded as independent prognostic risk factors. The m6AlRsPI was established based on the two m6A-lncRNAs as follows: (0.0006066 × expression level of LINC01820) + (0.0020769 × expression level of LINC02257). The cutoff of m6AlRsPI was 0.96. KM survival analysis for m6AlRsPI showed that the high m6AlRsPI group could contribute to higher mortality. The area under ROC curve for m6AlRsPI for predicting 3- and 5-year survival was 0.760 and 0.677, respectively, and the m6AlRsPI was also tested. The mutation and epithelial–mesenchymal transition (EMT) analysis for m6AlRsPI showed that the high m6AIRsPI group had more samples with gene mutation and had more likely caused EMT. Finally, gene ontology (GO) and Kyoto Encyclopedia of Genes and Genomes (KEGG) enrichment analysis were performed for mRNAs interacted with the two m6A-lncRNAs, showing they were involved in the process of RNA splicing and regulation of the mRNA surveillance pathway. qRT-PCR analysis showed that the two m6A-lncRNAs were upregulated in KIRC.

**Conclusion:** In the present study, hub m6A-lncRNAs were determined associated with metastasis in KIRC, and the ceRNA network demonstrated the potential carcinogenic regulatory pathway. Two m6A-lncRNAs associated with the overall survival were screened and m6AlRsPI was constructed and validated. Finally, the molecular signatures for m6AlRsPI and the two m6A-lncRNAs were analyzed to investigate the potential modulated processes in KIRC.

## Introduction

Renal cell carcinoma (RCC) is the third most common malignancy in the urogenital system (Siegel et al., 2019), with approximately 270,000 new diagnosed cases annually and 116,000 deaths around the world (Ljungberg et al., 2011). Among all renal tumors, 75% are kidney renal clear cell carcinoma (KIRC) (Rini et al., 2009), characteristically of high morbidity and mortality, for which chemotherapy, radiotherapy, and some other nonoperative treatments are difficult to obtain ideal outcomes (Rini et al., 2009). Patients with KIRC benefit majorly from surgery. There are still about 30% patients suffering from the risk of metastasis (Hsieh et al., 2017). Due to the complexity of carcinogenic regulatory network and the diversity of regulatory molecular modification, inhibition of known key targets cannot produce a marked effect. Other key factors that restrain tumor progression have become the major means for management of patients with metastatic KIRC. Therefore, it is crucial to identify other key regulatory factors for KIRC in an effort to improve the clinical outcome.

The accumulation of epigenetic alteration has become a momentous factor to advance development for tumors formatted by gene mutations. The aberrantly expressed long noncoding RNAs (lncRNAs) in specific cancers have been recognized as significant epigenetic regulatory molecules and as novel biomarkers for early diagnosis and therapy (Bach and Lee, 2018; Camacho et al., 2018). LncRNAs, defined as noncoding protein RNAs with more than 200 nucleotides are implicated in numerous bioprocesses of tumor cells, such as supporting proliferative signaling, eluding immune destruction and surveillance, capacitating replicative immortality, inducting angiogenesis, and activating invasion and metastasis (Gutschner and Diederichs, 2012; Bach and Lee, 2018). It has been found that upregulated TRPM2-AS contributes to tumor cell proliferation through TRPM2 stabilized by TAF15 in colorectal cancer (Pan et al., 2020). Qin et al. (2019) demonstrated that lncRNA MIR155HG acted as a tumor-promoting factor through inhibition of miR-802 that is a tumor suppressor in pancreatic cancer, providing a potential diagnostic and therapeutic target. Also in ovarian cancer, lncRNA HOTTIP boosts the expression of IL-6 to lead the immune escape of tumor cells by upregulating PD-L1 expression in neutrophils (Shang et al., 2019). In addition, the occurrence of tumor resistant to chemotherapy can also be caused by dysregulated lncRNA H19 through modulation of the glutathione metabolism pathway in advanced ovarian cancer (Zheng et al., 2016). Therefore, the regulatory role of lncRNAs to tumor is not less than that of oncogene or tumor suppressor gene, elucidating the significance of the landscape of lncRNAs in tumors.

N6-methyladenosine (m6A) refers to methylation at the N6 position of adenosine, which has been demonstrated as the most ubiquitous, abundant, and conserved internal dynamic posttranscriptional chemical modification within mRNA (Meyer and Jaffrey, 2014; Zhao et al., 2017). Functionally, m6A modulates mRNA maturation, splicing, translation, expression, and degradation and other major bioprocesses that have not been discovered yet (Meyer and Jaffrey, 2014; Zhao et al., 2017). It is not so surprising that accumulating evidence demonstrated that m6A was implicated in regulating hallmarks of tumors with tumorigenesis, proliferation, differentiation, invasion, and metastasis (Lin et al., 2016; Chen et al., 2019; Wang T. et al., 2020). The reversible processes of m6A modification were catalyzed by methyltransferases (termed as “writers”), demethylating enzymes (termed as “erasers”), and m6A-binding proteins (termed as “readers”) (Meyer and Jaffrey, 2014). METTL3, classified as writers, inhibits SOX2 degradation for conducting the progression of colorectal carcinoma in an m6A-IGF2BP2–dependent manner (Li et al., 2019) and was linked with poor prognosis of gastric cancer due to METTL3-mediated HDGF of methylation facilitating cancer progression (Wang Q. et al., 2020). ALKBH5, an m6A demethylase, is capable of attenuating WIF-1 RNA methylation and mediating the Wnt signaling pathway, to restrain the progression of pancreatic cancer, causing a favorable clinical outcome for patients (Tang et al., 2020). Readers are the factors that execute functions. YTHDF2, classified as readers, causes liver cancer metastasis by elevating the expression of OCT4 in an m6A RNA methylation manner (Zhang et al., 2020). Therefore, m6A modification offers a novel insight into the interplay between mRNAs and cancers.

m6A modification also occurs within lncRNAs, with the equally significant role in regulating bioprocesses of tumor (Yi et al., 2020). lncRNA NEAT1-1 with m6A modification is capable to regulate the Pol II ser2 phosphorylation and promotes the formation of the complex CYCLINL1/CDK19/NEAT1-1 contributing to bone metastasis of prostate cancer (Wen et al., 2020). In colorectal cancer, lncRNA LINRIS modulates the expression of MYC to influence the process of glycolysis, leading to the progression of cancer by stabilizing IGF2BP2 (Wang H. et al., 2019). However, the hub lncRNAs with m6A modification (m6A-lncRNAs) that are associated with the prognosis in KIRC remain unclear. The present study aims to identify m6A-lncRNAs in KIRC and investigate their biological signatures and prognostic values, providing promising targets for further research.

## Materials and Methods

### Acquisition of Data About KIRC

The lncRNA-seq profiles, miRNA-seq profiles, and mRNA-seq profiles of KIRC samples and corresponding clinical characteristics including survival time, status, age, gender, grade, and TNM staging were downloaded from The Cancer Genome Atlas (TCGA) (https://portal.gdc.cancer.gov/). External cohort (GSE40914) was gained from Gene Expression Omnibus (GEO) (https://www.ncbi.nlm.nih.gov/geo/query/acc.cgi) to validate the prognostic value of screened m6A-lncRNA. The identified genes with m6A modification were retrieved from the RMVar database (http://rmvar.renlab.org/index.html) (Luo et al., 2020). Data with missing or unknown values were deleted. Then, we randomly and equally divided the parental TCGA cohort into two subgroups. There was no discrepancy between them. A subgroup was regarded as the training dataset and another as the testing dataset.

### Acquirement of the Hub m6A-lncRNAs

#### Production of Gene Modules

In the training dataset, the R package “edgeR” was utilized to perform differentially expressed analysis on the lncRNA-seq data in R software (version 4.0.2), with the statistical significance of | log2 fold change (FC) | > 1 and the false discovery rate (FDR) < 0.05. After intersecting DElncRNAs with m6A-mediated genes, the weighted gene co-expression network analysis (WGCNA) was conducted to identify hub m6A-lncRNAs in KIRC. First, the Pearson’s correlation coefficient of the pairwise m6A-lncRNAs was calculated for constructing the similarity matrix. The similarity matrix was transformed into a weighted adjacency matrix after a soft threshold of β was identified. Next, the adjacency matrix was transformed into topological overlap matrix (TOM) measuring the connectivity between genes. Then, in terms of dissimilarity measure (1-TOM), average linkage hierarchical clustering was performed to cluster the genes with similar expression profiles for producing gene modules. The dissimilarity of module eigengenes (MEs) was calculated.

#### Identification of Significant Modules Linked With Clinical Traits

Gene significance (GS) obtained from the log10 transformation of the *p* value in a linear regression between the gene expression and clinical traits was computed to assess the significance of each module. Then, the average GS within a module was called as module significance (MS), which can be used to analyze the association between a module and clinical characteristics. The module correlated to a clinical trait was the one with the largest MS among all selected modules. The correlation between the MEs and clinical characteristics including grade and TNM staging was also calculated to determine the relevant module associated with clinical information. These processes were called as module–trait relationship (MTR) analysis (Langfelder and Horvath, 2008).

#### Obtaining Hub m6A-lncRNAs

A network was constructed according to the edges between two m6A-lncRNAs with weight >0.5 after screening m6A-lncRNAs correlated with clinical modules (the red and turquoise modules). On the basis of the plug-in unit “cytoHubba” within the Cytoscape software (version 3.7.2), m6A-lncRNAs in the network with Maximal Clique Centrality (MCC) ≥ 2 were identified as the hub m6A-lncRNAs.

### Construction of an lncRNA-miRNA-mRNA Network Based on Hub m6A-lncRNAs

The R package “edgeR” was applied to conduct a differentially expressed analysis on the miRNA-seq and mRNA-seq of KIRC samples in R software. The selection criteria were the same as described previously. Then, weighted correlation network analysis (WGCNA) was performed on hub m6A-lncRNAs-DEmiRNAs profile to calculate the connectivity between molecules. The analysis process was described previously. An optimal soft threshold of β was equal to three. The edges between hub m6A-lncRNAs and DEmiRNAs with a threshold weight >0.3 were selected. Next, WGCNA was also performed on DEmiRNAs–DEmRNAs profile. The soft threshold of β was set to five. The edges between DEmiRNAs and DEmRNAs with a threshold weight >0.3 were opted. Finally, took the DEmiRNAs as a bridge, an lncRNA-miRNA-mRNA (ceRNA) network was constructed and visualized by the Cytoscape software (version 3.7.2). The Kaplan–Meier (KM) survival analysis along with the log-rank test on the regulatory molecules in the ceRNA network was performed.

### Development of m6A-lncRNAs Prognostic Index

The univariate Cox regression analysis was conducted on the hub m6A-lncRNAs to screen the m6A-lncRNAs associated with overall survival. Based on the results of the univariate analysis, the multivariate Cox regression analysis was utilized to identify the independent prognostic factors. The m6A-lncRNAs prognostic index was constructed with the hub m6A-lncRNAs of *p* < 0.05 in the multivariate Cox regression model through multiplying the expression values of them by their coefficient in the model and then adding them together. The patients were then divided into high-m6AlRsPI and low-m6AlRsPI groups based on the median m6AlRsPI value. After screening the hub m6A-lncRNAs correlated with the overall survival through the Cox regression analysis, we performed the KM survival analysis on the relevant m6A-lncRNAs with statistical significance and the m6AlRsPI to estimate their prognostic power. The performance of m6AlRsPI for predicting 3- and 5-year overall survival of KIRC patients was analyzed by receiver operating characteristic (ROC) curve.

### Validation for m6AlRsPI

Internal and external cohorts were applied to validate the prognostic signature of m6AlRsPI. In the testing dataset, the analysis processes were described previously. The m6AlRsPI was constructed and the prognostic and predictive signatures for m6AlRsPI in the testing cohort were estimated. External cohort (GSE40914) was also utilized to verify prognostic values of screened m6A-lncRNAs.

### Analysis of Gene Mutation in Different m6AlRsPI Subgroups

The mutation analysis for KIRC samples was investigated to display the discrepant gene mutation in different m6AlRsPI subgroups. The major mutant genes and variant classifications in different m6AlRsPI subgroups were our concern.

### Association of m6AlRsPI Subgroups With Epithelial–Mesenchymal Transition

The correlation between different m6AlRsPI subgroups and epithelial–mesenchymal transition (EMT) was explored to show the prognostic power in different m6AlRsPI subgroups, by analyzing the expression of CDH1, VIM, SNAI1, SNAI2, and CHD2 in high m6AlRsPI and low m6AlRsPI groups. The expression of PDCD1 in different m6AlRsPI subgroups was also calculated.

### Molecular Signature Analysis for Screened Prognosis-Associated Hub m6A-lncRNAs

We looked for RNA-binding proteins (RBPs) in The Encyclopedia of RNA Interactomes (ENCORI) (http://starbase.sysu.edu.cn/index.php), RBPDB database (http://rbpdb.ccbr.utoronto.ca/), and catRAPID database (http://s.tartaglialab.com/page/catrapid_group) to obtain mRNAs interacted with the screened prognosis-associated hub m6A-lncRNAs. Then, Gene Ontology (GO) and Kyoto Encyclopedia of Genes and Genomes (KEGG) enrichment analyses were conducted to exhibit the potential regulatory signaling pathways in KIRC.

### Validation of the Expression Level of Screened Hub m6A-lncRNAs in KIRC by qRT-PCR

Total RNA was extracted from cell lines using TRIzol reagent (Invitrogen, China) in terms of the manufacturer’s protocol. HK-2 is a normal kidney cell line, whereas ACHN, 769-P, and 786-O are KIRC cell lines. Reverse transcription was conducted on RNA into cDNA with RevertAid First Strand cDNA Synthesis Kit (Thermo Scientific; United States) according to the manufacturer’s guidelines. Quantitative reverse transcription–polymerase chain reaction (qRT–PCR) was performed using FastStart Universal SYBR Green Master (ROX) (Roche; United States). GAPDH was used as the internal control and the relative expression levels of m6A-lncRNA were calculated by 2^−ΔΔCt^. The primer sequences utilized in this study were as follows: GAPDH-F: GGA​AGG​TGA​AGG​TCG​GAG​TCA, GAPDH-R: GTC​ATT​GAT​GGC​AAC​AAT​ATC​CAC​T; LINC01820-F: GGC​CCA​CCC​ACA​TAG​TTT​AAA​GCC​A, LINC01820-R: GCA​CAC​TCA​CAG​AAC​GCA​AA; LINC02257-F: AGG​TGG​AGT​CTC​GCA​CTG​TCA​TCC​T, LINC02257-R: TTC​ACT​GGT​TTG​CTC​TGC​AAT​CCC​A.

## Statistical Analysis

The continuous variables were compared by independent *t*-test. Univariate and multivariate prognostic analysis were performed in the Cox regression model. The survival analysis was performed by the Kaplan–Meier survival analysis with the log-rank test. The R package “survival ROC” was utilized to plot the time-dependent ROC curve. The threshold of statistical significance was *p* < 0.05.

## Results

### Obtaining m6A-lncRNAs for KIRC

**FIGURE 1:**
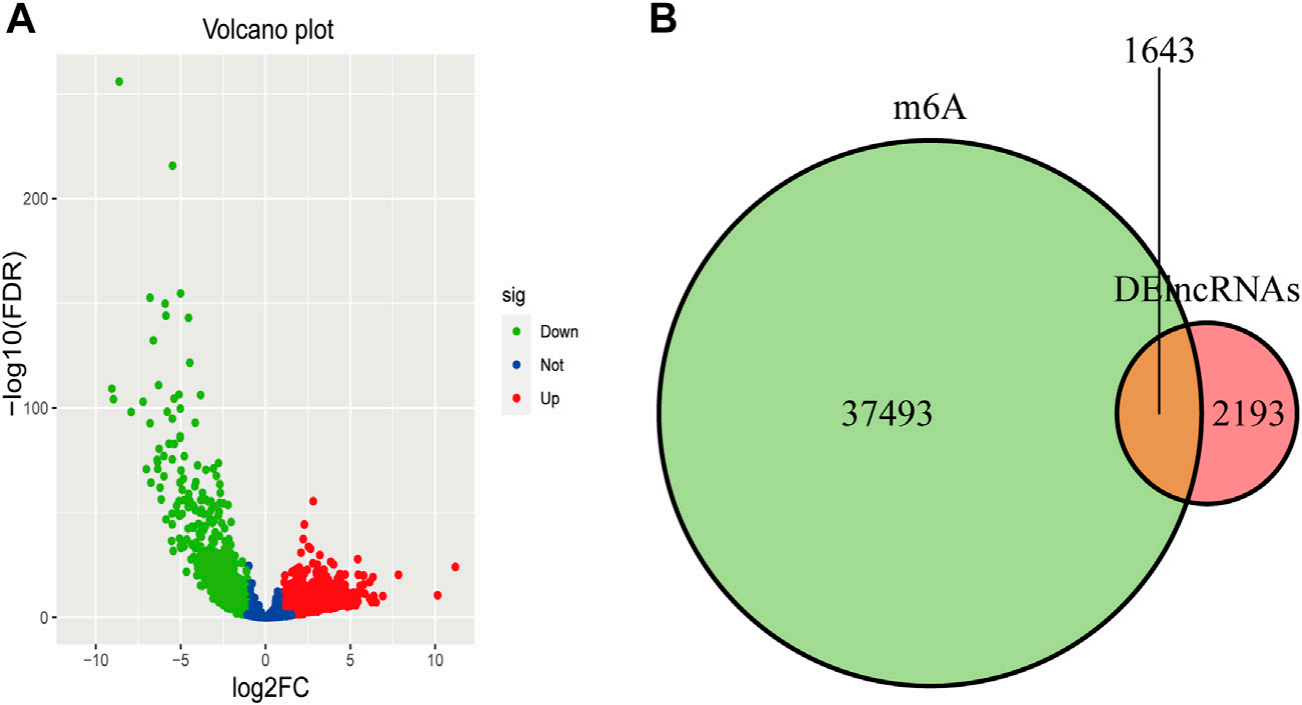
Differentially expressed m6A-lncRNAs. **(A)** The volcano plot has shown the differentially expressed lncRNAs in the training cohort in KIRC. The green dots, blue dots, and red dots mean the downregulated, no differentially expressed, and upregulated lncRNAs, respectively. **(B)** The intersection of DElncRNAs with m6A-modified genes was illustrated by the Venn diagram.

Differentially expressed analysis performed on the training dataset (271 tumors vs. 35 normal samples), the sum of DElncRNAs was 3,836, including 1,221 downregulated lncRNAs and 2,615 upregulated lncRNAs, as shown in Figure 1A. A total of 1,643 differentially expressed m6A-lncRNAs were identified following intersection of 3,836 DElncRNAs with 39,136 m6A-modified genes, as shown in Figure 1B. After clearing invalid data, the data of clinical characteristics are presented in Supplementary Table S1.

### Identification of Hub m6A-lncRNAs Related to Clinical Traits

**FIGURE 2:**
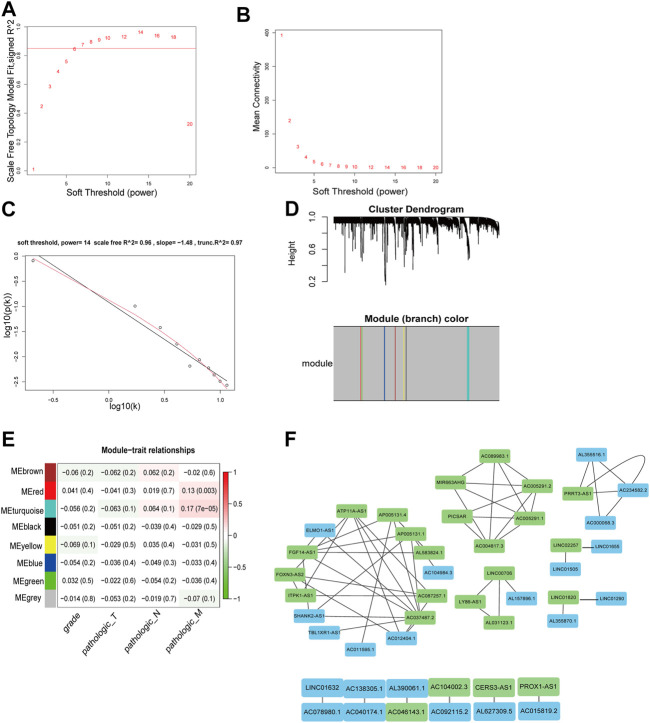
The processes of screening the hub m6A-lncRNAs. **(A)** Analysis of the scale-free fit index for various soft thresholds, and the soft threshold was set as 14. **(B)** Analysis of the mean connectivity for various soft thresholds. **(C)** Checking the scale-free topology when the soft threshold = 14. **(D)** Dendrogram showing the clustered m6A-DElncRNAs. **(E)** Heat map illustrating the association of the MEs with clinical characteristics (including grade and TNM staging). The modules with *p* < 0.05 were selected. **(F)** Network for m6A-lncRNAs within the red and turquoise modules. The blue boxes mean hub-lncRNAs within the red module and the green boxes represent hub-lncRNAs within the turquoise module. And lines indicate that there is a correlation between m6A-lncRNAs.

In the training cohort, WGCNA was conducted on 1,643 differentially expressed m6A-lncRNAs to obtain the hub m6A-lncRNAs in KIRC. The optimal soft threshold value was selected as 14 (scale free *R* ^2^ = 0.96) to ensure a scale-free network (Figures 2A,B). We further analyzed the reliability of the scale-free topology in the light of soft threshold equal to 14 (Figure 2C). The logarithm log10 (k) of the node with connectivity K was negatively associated with the logarithm log10 [*p*(k)] of the probability of the node. According to the similar expression pattern, a total of eight modules were determined by average linkage clustering (Figure 2D). Red and turquoise modules were found to have a correlation with tumor M stage by MTR analysis (Figure 2E). There were 22 m6A-lncRNAs within red module and 25 m6A-lncRNAs within turquoise module, with a threshold weight >0.5. Then, m6A-lncRNAs within the two modules were put into the Cytoscape software, to construct a network (Figure 2F) and to calculate the MCC value which is listed in Supplementary Table S2, and m6A-lncRNAs with MCC ≥2 were identified as the hub m6A-lncRNAs. Finally, the total of hub m6A-lncRNAs was equal to 21.

### Construction of an lncRNA-miRNA-mRNA Network

**FIGURE 3:**
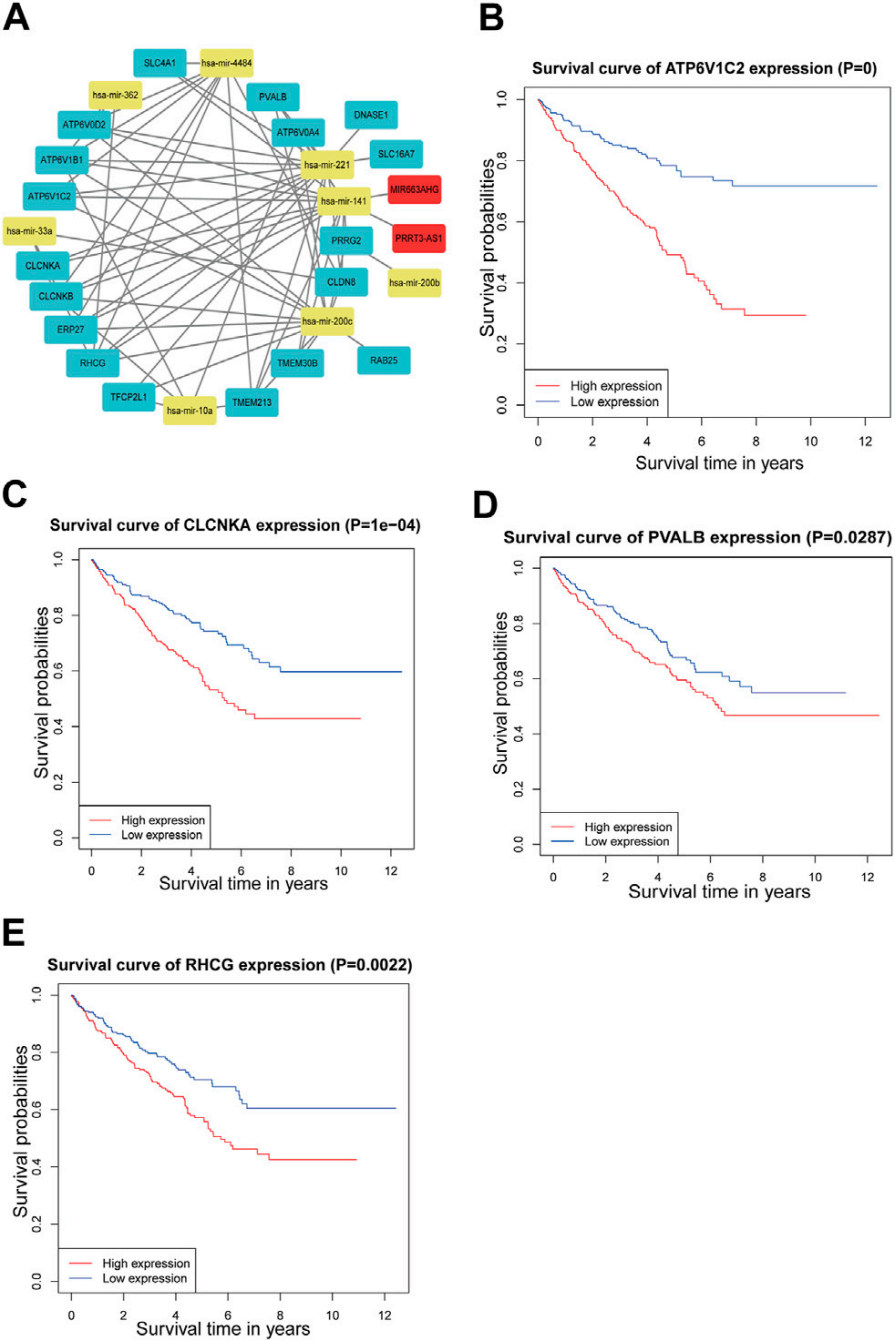
ceRNA network and survival analysis. **(A)** ceRNA network based on the hub m6A-lncRNAs. There were two hub m6A-lncRNAs, eight DEmiRNAs, and 18 DEmRNAs in the ceRNA network. The red squares represent hub m6A-lnRNAs, yellow squares are miRNAs, and blue squares mean mRNAs. **(B–E)** The survival analysis for ATP6V1C2, CLCNKA, PVALB, and RHCG.

After performing differential expression analysis on the miRNA-seq and mRNA-seq for KIRC samples, a total of 173 DEmiRNAs and 722 DEmRNAs were obtained, respectively. First, WGCNA was conducted on the hub m6A-lncRNA-DEmiRNAs profile to determine 19 hub m6A-lncRNAs associated with 107 DEmiRNAs. Similarly, 77 DEmiRNAs correlated with 459 DEmRNAs were obtained by performing WGCNA on DEmiRNAs-DEmRNAs profile. Regarding the common DEmiRNAs on both profiles as the connection point, the lncRNA-miRNA-mRNA network (ceRNA network) comprising of two hub m6A-lncRNAs, eight DEmiRNAs, and 18 DEmRNAs was constructed (Figure 3A). The potential regulatory pathways based on ceRNA network contain MIR663AHG/hsa-mir-141/SLC4A1 etc. and PRRT3-AS1/hsa-mir-141/SLC4A1 etc. Then, other lncRNA-miRNA correlation networks and miRNA-mRNA correlation networks are shown in Supplementary Figures S1A,B. The survival analysis on the molecules within the ceRNA network was conducted. However, the two hub m6A-lncRNAs (MIR663AHG and PRRT3-AS1) and miRNA (has-mir-141) had no impact on the overall survival of patients with KIRC (Supplementary Figures S2A–C). In addition, four mRNAs (ATP6V1C2, CLCNKA, PVALB, and RHCG) were found to be associated with the prognosis for patients (Figures 3B–E). Other mRNAs with no survival difference are shown in Supplementary Figures S2D–M.

### Establishment of m6A-lncRNAs Prognostic Index Based on Cox Regression Analysis

**FIGURE 4:**
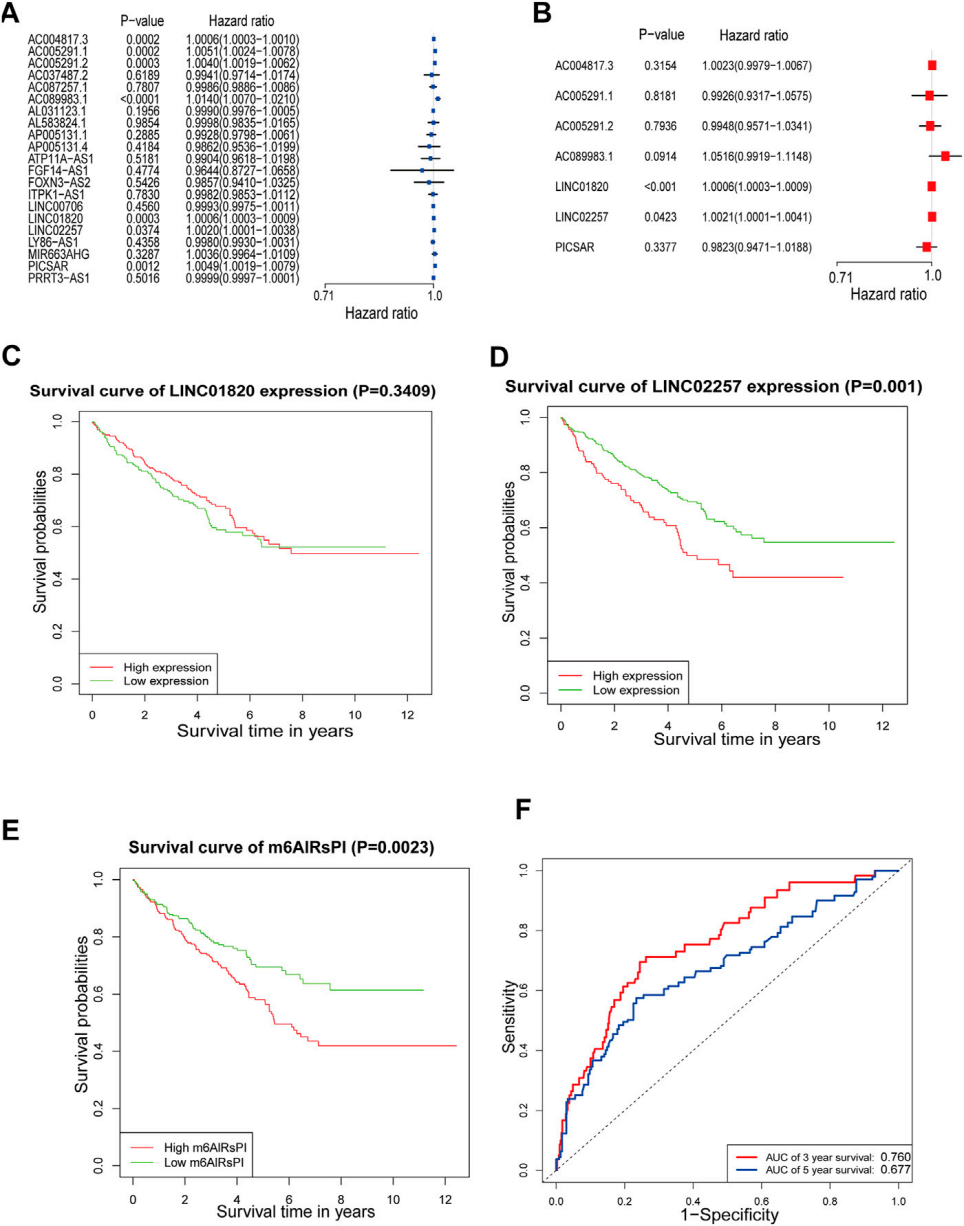
Cox regression analysis and survival analysis. **(A)** The result of univariate Cox regression analysis for m6A-lncRNAs in red and turquoise modules shown by forest plot. **(B)** The outcome of multivariate Cox regression analysis for m6A-lncRNAs with *p* < 0.05 in univariate analysis. **(C–E)** Survival analysis for LINC01820, LINC02257, and m6AlRsPI in the training cohort **(F)** ROC curve for m6AlRsPI for predicting 3- and 5-year survival. The red curve means 3-year survival prediction, with the area under the curve of 0.760. The blue curve means 5-year survival prediction, with the area under the curve of 0.677.

The Cox regression analysis was conducted on the 21 hub m6A-lncRNAs to screen the prognostic factors for patients with KIRC. The outcome of the univariate Cox regression analysis was illustrated in Figure 4A. The hub m6A-lncRNAs with statistical significance were included in the multivariate Cox regression analysis (Figure 4B). It was found that LINC01820 (HR: 1.0006; 95% CI: 1.0003–1.0009; *p* < 0.001) and LINC02257 (HR: 1.0021; 95% CI: 1.0001–1.0041; *p* = 0.0423) were closely correlated with the poor prognosis of patients with KIRC (risky hub m6A-lncRNAs). The m6A-lncRNAs prognostic index (m6AlRsPI) was established based on the two m6A-lncRNAs as follows: m6AlRsPI = (0.0006066 × expression level of LINC01820) + (0.0020769 × expression level of LINC02257). The median value of m6AlRsPI was 0.96, as the cutoff to separate KIRC samples into the high and the low m6AlRsPI group. The Kaplan–Meier survival analysis for the two hub m6A-lncRNAs and m6AlRsPI in the training cohort were carried out to investigate the association with the overall survival. The results demonstrated that the high expression level of LINC02257 had an unfavorable overall survival as compared with low expression level of LINC02257 (Figure 4D). However, there was no survival discrepancy between low expression level and high expression level of LINC01820 (Figure 4C). The high m6AlRsPI group could contribute to higher mortality than that of the low m6AlRsPI group (Figure 4E). The area under ROC curve for m6AlRsPI for predicting 3- and 5-years survival was 0.760 and 0.677, respectively Figure 4F, demonstrated a moderate performance for predictive prognosis.

### Validation of the Reliability for the m6AlRsPI

**FIGURE 5:**
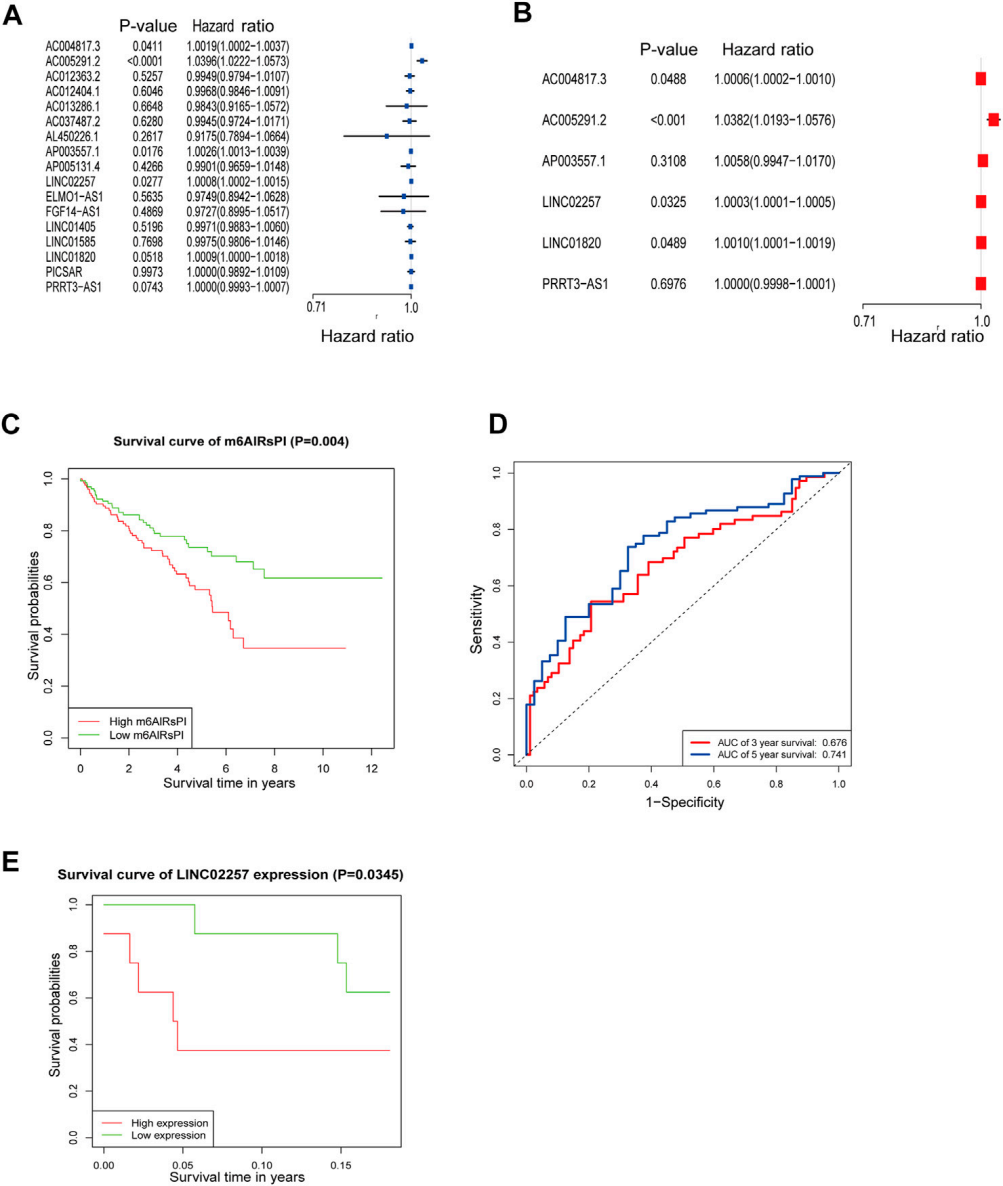
Internal and external validation for m6AlRsPI. **(A)** The result of univariate Cox regression analysis for m6A-lncRNAs associated with M stage in gray module in the testing cohort, shown by forest plot. **(B)** The outcome of multivariate Cox regression analysis for hub lncRNAs with *p* < 0.05 within the univariate Cox regression analysis. **(C)** KM survival analysis for m6AlRsPI constructed by testing cohort. **(D)** In testing cohort, ROC curve for predicting 3- and 5-year survival. **(E)** Validation of the survival signature for LINC02257 within GEO dataset.

For internal validation, the testing cohort was used to validate the m6AlRsPI model developed by the training cohort (Supplementary Table S1). The m6A-lncRNAs in the testing cohort were screened by WGCNA as described previously (Supplementary Figures S3A–D). The hub m6A-lncRNAs with *p* < 0.05 within the univariate Cox regression analysis were taken into multivariate Cox regression analysis (Figures 5A,B) to identify hub m6A-lncRNAs correlated with prognosis in the testing cohort. Then, the m6AlRsPI model based on LINC02257 and LINC01820 was also constructed, and the KM survival analysis showed high m6AlRsPI group with poor prognosis (Figure 5C). Moreover, in the testing cohort, the area under ROC curve for predicting 3- and 5-years survival was 0.676 and 0.741, respectively (Figure 5D), with moderate predictive power, consistent with the result above. The KM survival analysis for LINC02257 within GEO cohort demonstrated a high expression level of LINC02257 which increased the mortality (Figure 5E) and no relevant external dataset was found to validate the prognostic value of LINC01820.

### Gene Mutation Analysis for m6AlRsPI Subgroups

**FIGURE 6:**
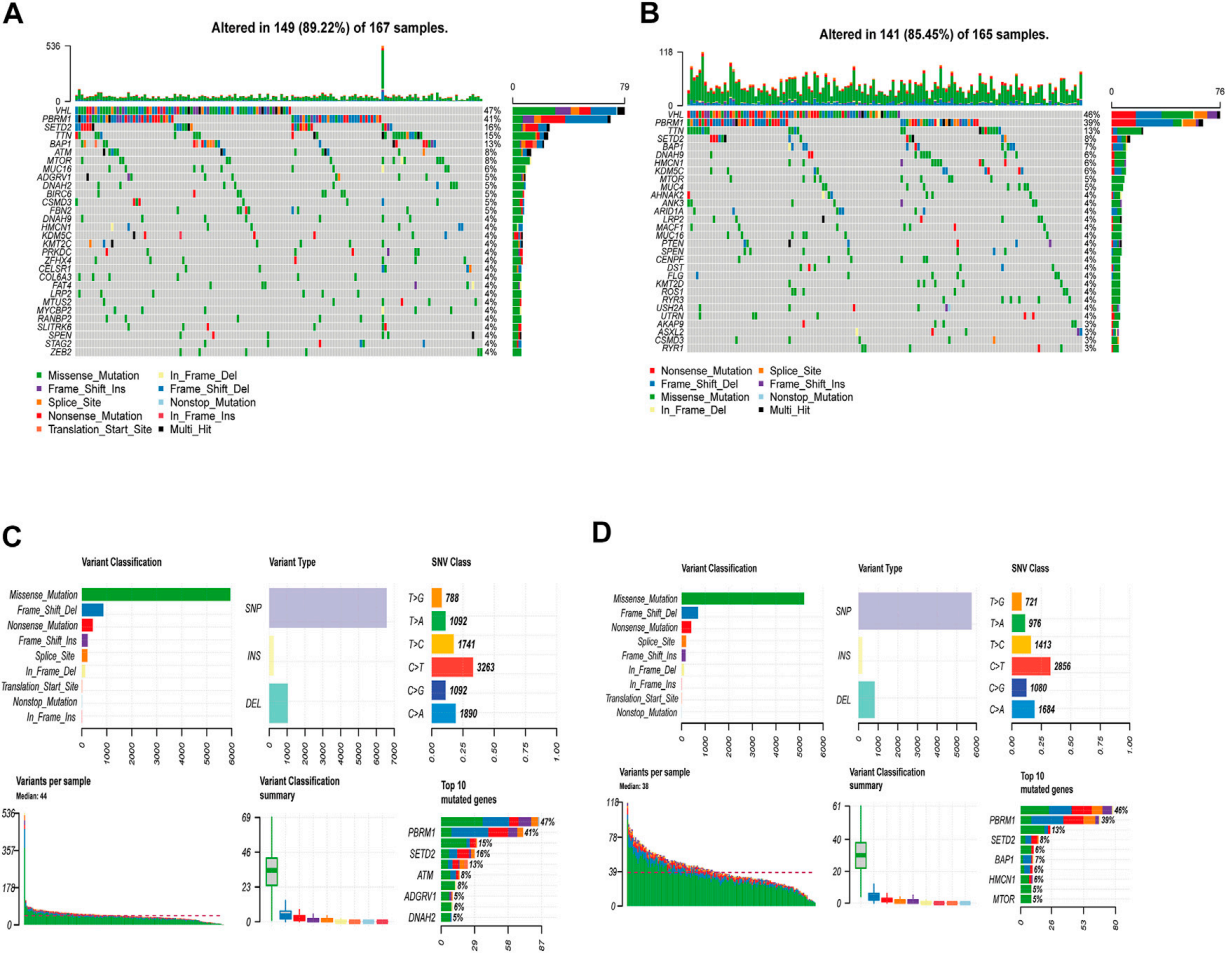
Mutation analysis based on m6AlRsPI subgroups. **(A)** The waterfall plot demonstrated mutational genes in high m6AlRsPI group. **(B)** Mutational genes in low m6AlRsPI group. **(C)** Summary for mutation in high m6AlRsPI group. **(D)** Summary for mutation in low m6AlRsPI group.

The gene mutation was analyzed to gain a novel insight into the molecular nature in m6AlRsPI subgroups. The gene mutation frequency in the high m6AlRsPI group was higher than that of the low m6AlRsPI group (89.22 vs. 85.45%). The top 10 mutational genes in the high m6AlRsPI group contain VHL, PBRM1, SETD2, TTN, BAP1, ATM, MTOR, MUC16, ADGRV1, and DNAH2 (Figure 6A). The mutation rate of more than 10% genes includes VHL, PBRM1, SETD2, TTN, and BAP1. The top 10 mutational genes in the low m6AlRsPI group comprise of VHL, PBRM1, TTN, SETD2, BAP1, DNAH9, HMCN1, KDM5C, MTOR, and MUC4 (Figure 6B). VHL, PBRM1, and TTN are the genes with the mutation rate of more than 10% genes. The most common mutation type in both groups is missense-mutation, followed by frameshift deletions and nonsense mutation (Figures 6C,D).

### Association of Epithelial–Mesenchymal Transition With m6AlRsPI Subgroups

**FIGURE 7:**
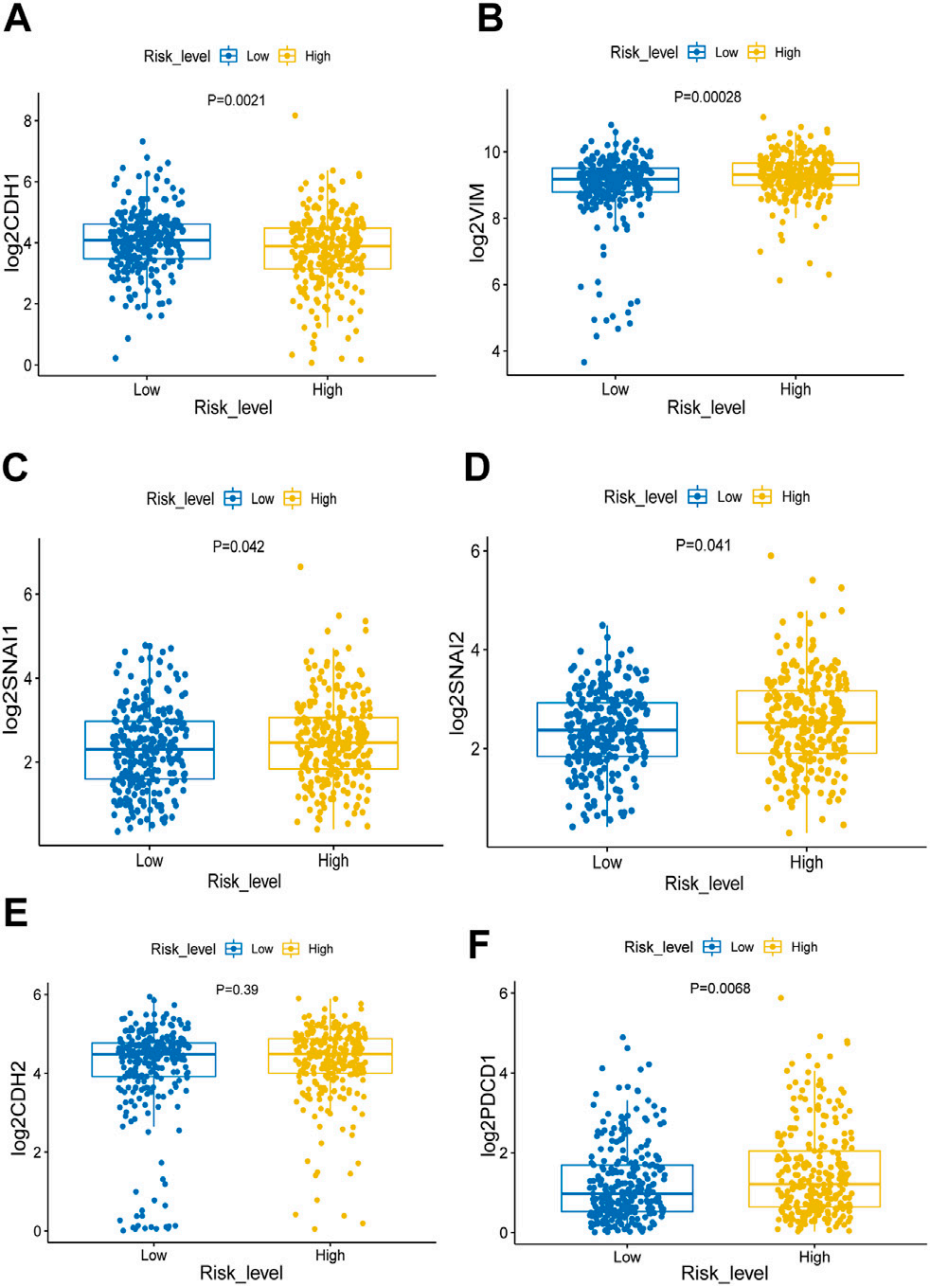
Analyzing association with EMT and PDCD1 for m6AlRsPI subgroups. **(A)** The expression level of CDH1 in high m6AlRsPI group was decreased as compared with the low m6AlRsPI group. **(B–E)** Genes correlated with mesenchyme in high m6AlRsPI group were upregulated expression. **(F)** The expression level of PDCD1 was upregulated in high m6AlRsPI.

As the hub m6A-lncRNAs are closely linked with the clinical trait of M stage, we made further analysis of the association of EMT with m6AlRsPI subgroups. E-cadherin is regarded as epithelial biomarker, which is coded by CDH1 gene. It was found that in the high m6AlRsPI group, the expression level of CDH1 gene decreased as compared with the low m6AlRsPI group (Figure 7A). Mesenchymal biomarkers include N-cadherin, vimentin, SNAI1, and SNAI2. N-cadherin and vimentin are coded by CDH2 and VIM gene, respectively. The analysis results showed the expression level of VIM, SNAI1, and SNAI2 in the high m6AlRsPI group were higher than that in the low m6AlRsPI group (Figures 7B–D). There was no statistical difference for the expression of CDH2 between the two groups (Figure 7E). In addition, the expression of PDCD1 between the two groups is statistically different (Figure 7F).

### GO and KEGG Enrichment Analysis for Proteins Interacted with the Two Hub m6A-lncRNAs

**FIGURE 8:**
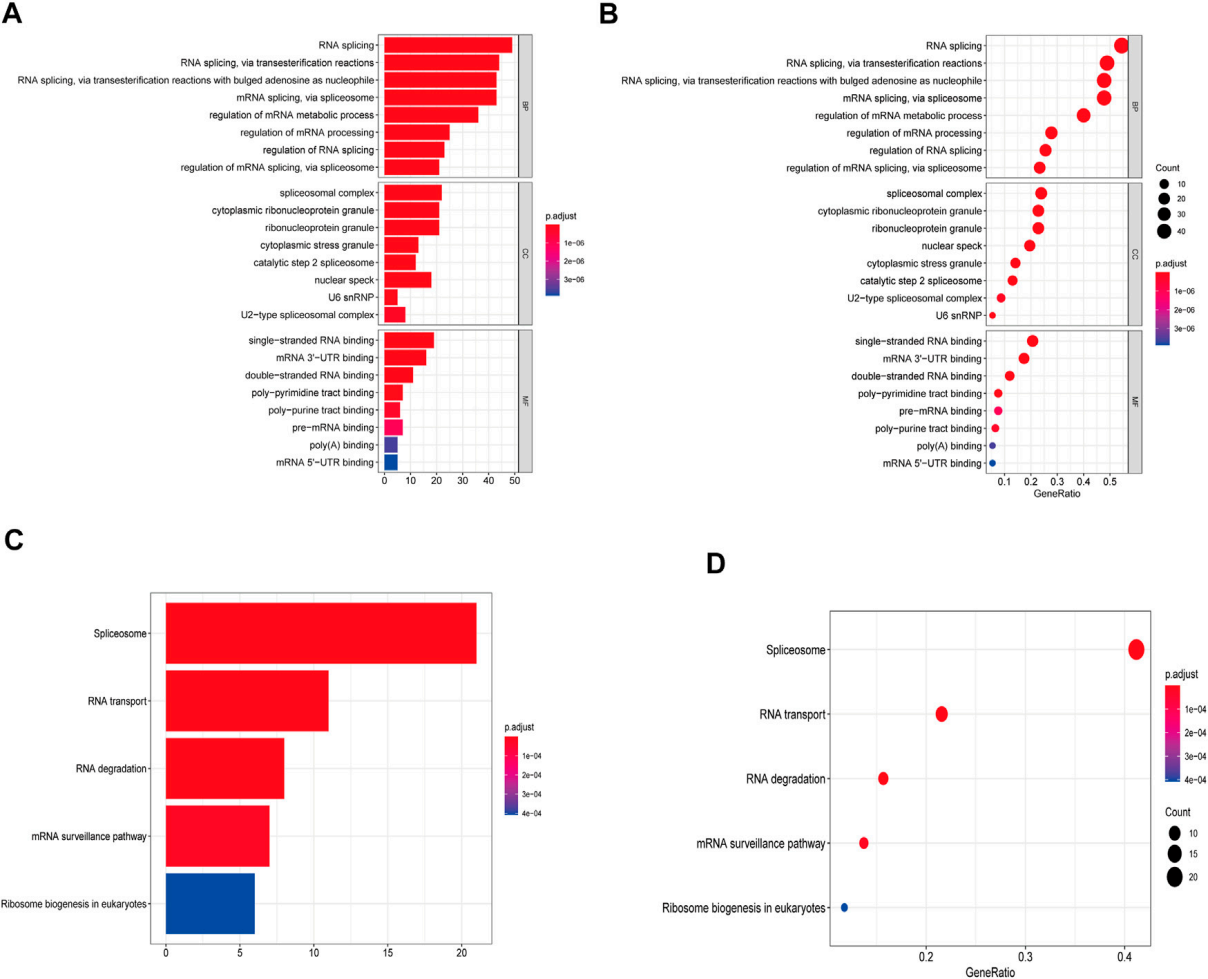
GO and KEGG analysis. **(A,B)** The bar plot and dot plot of GO analysis for the top eight enrichment. **(C,D)** The bar plot and dot plot of KEGG analysis for the top five enrichment.

After looking for RNA-binding proteins (RBPs) in The Encyclopedia of RNA Interactomes (ENCORI), RBPDB database, and catRAPID database, a total of 153 mRNAs interacted with the two hub m6A-lncRNAs were identified for GO and KEGG enrichment analysis. The RBP-lncRNA interaction in ENCORI was supported by CLIP-seq Data, and in RBPDB and catRAPID omics was predicted by RNA sequencing. The outcome from the GO analysis showed they were involved in processes of RNA splicing and mRNA metabolism (Figures 8A,B). KEGG analysis demonstrated they are available to modulate RNA transport and degradation and mRNA surveillance signaling pathway (Figures 8C,D).

### The Expression Levels of Hub m6A-lncRNAs in KIRC

**FIGURE 9:**
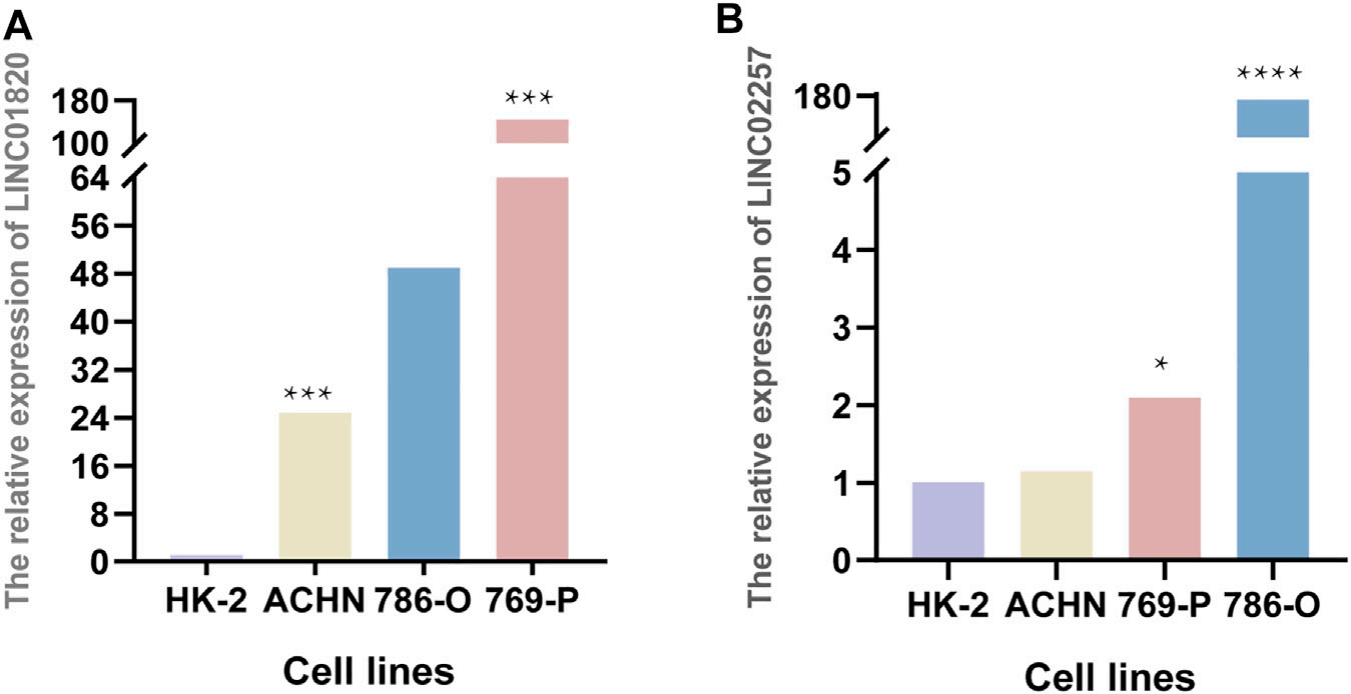
Quantitative analysis for LINC01820 and LINC02257. **(A,B)** The expression levels of LINC01820 and LINC02257 in HK-2, ACHN, 786-O, and 769-P cell lines. *p < 0.05, ***p < 0.001, ****p < 0.0001.

The expression levels of hub m6A-lncRNAs in KIRC were quantified by qRT-PCR. Compared to the normal kidney cell line (HK-2), the expression level of LINC01820 was elevated in KIRC cell lines (ACHN and 769-P), with statistical significance (Figure 9A). The expression level of LINC02257 was upregulated in 769-P and 786-O cell lines (Figure 9B). These results were consistent with our analysis results based on TCGA KIRC cohort, showing LINC01820 and LINC02257 were significant molecules in modulating KIRC progression.

## Discussion

Metastatic KIRC is the major cause of poor clinical outcome and higher mortality for patients with kidney cancer. Although traditional targeted therapy and novel immunotherapy were utilized to treat patients with metastatic KIRC, no satisfactory lasting responses of drugs to tumors and 5-year overall survival rate for patients could not be obtained. For those patients who have lost the opportunity of surgery, inhibition of key regulatory targets for metastasis may be the most effective therapy (Thomas and Kabbinavar, 2015). Due to discovery of implicating in the diverse considerable bioprocesses for tumor, lncRNAs are regarded as a novel regulatory factor for investigating the mechanism of cancer metastasis (Gutschner and Diederichs, 2012). A variety of evidence has manifested the pivotal role of lncRNAs in regulating tumor metastasis. LncRNA MALAT1 has previously been regarded as a metastasis-promoting factor in various tumors, but it is a metastasis-suppressing factor in breast cancer through binding and inactivating the pro-metastatic transcription factor TEAD (Kim et al., 2018). LncRNA RP11-390F4.3 is induced by hypoxia/HIF-1α to facilitate EMT through modulation of multiple EMT-associated factors, leading to tumor metastasis (Peng et al., 2020). In addition, m6A has been identified as the most common chemical modification manner for various RNAs in eukaryotic cells, and it is involved in the progression of tumor through relative key regulatory factors with m6A modification (Wang Q. et al., 2020). The brain metastasis of lung cancer results from m6A-mediated matured miR-143-3p upregulating the expression of VASH1 to increase ubiquitylation of VEGFA (Wang Y. et al., 2019). BATF2 with m6A modification suppresses gastric tumor cell metastasis by stabilizing *p*53 protein to inhibit phosphorylation of ERK, causing a favorable prognosis (Xie et al., 2020). Furthermore, the process of EMT correlated with tumor metastasis can be triggered by m6A-related molecules. Li et al. (2020) demonstrated the overexpression of METTL3 induced EMT of cancer cells through modulation of expression and secretion of TGFβ1. Hua et al. (2018) found METTL3 promoted EMT by upregulating the receptor tyrosine kinase AXL in ovarian cancer. The crucial regulatory role of m6A modification in tumor metastasis ought to be paid attention. Therefore, the hub m6A-lncRNAs are vital targets for diagnosis, surveillance, and therapy of metastatic tumor, including KIRC. In colorectal cancer, m6A methylation can induce the upregulation of lncRNA RP11 to modulate Siah1-Fbxo45/Zeb1 complex to promote the dissemination of tumor cells (Wu et al., 2019). Zheng et al. (2019) demonstrated lncRNA FAM225A is an upregulated oncogenic lncRNA in nasopharyngeal carcinoma and m6A-mediated lncRNA FAM225A gives rise to tumor metastasis by sponging miR-590-3p and miR-1275 to upregulate ITGB3 and activate the FAK/PI3K/Akt signaling pathway. m6A-mediated LINC00470 facilitates gastric cancer cell distant metastasis by accelerating degradation of PTEN mRNA (Yan et al., 2020). In cervical cancer, lncRNA ZFAS1 with m6A modification conduces to unfavorable clinical outcome by suppressing miR-647 to lead to tumor metastasis (Yang et al., 2020). Currently, the significant biomarkers correlated with metastatic KIRC and relative regulatory mechanism have remained unknown. It is urgent to look for effective factors implicated in the modulation of metastasis to improve the overall survival of patients with KIRC. Additionally, hub m6A-lncRNAs can be regarded as extremely important factors for investigating the complex mechanism of metastasis as a result of abundant regulation functions.

Based on WGCNA, DElncRNAs with m6A modification were divided into modules with the correlation analysis. After calculating the correlation between modules and clinical characteristics, we found two modules that were linked with the clinical trait of M stage. Although they were weakly correlated, the hub m6A-lncRNAs with a potential regulatory role were of interest. Therefore, m6A-lncRNAs within the two modules were considered related with metastasis of KIRC and might be treated as potential major molecules for modulating tumor metastasis. Then, construction of a ceRNA network aims to investigate the potential regulation pathways of 21 hub m6A-lncRNAs. Cytoscape software was utilized to screen the hub m6A-lncRNAs, and the Cox regression analysis was performed to identify hub m6A-lncRNAs correlated closely with the prognosis. However, prognosis-associated m6A-lncRNAs were not embodied in the ceRNA network. The m6A-lncRNAs within the ceRNA network were not correlated with overall survival by the KM survival analysis and Cox regression analysis. So it was shown that prognosis-associated m6A-lncRNAs may influence biological behaviors of KIRC through other manners (such as RNA-binding proteins manner) instead of lncRNA-miRNA-mRNA manner. Identified LINC02257 was shown to be associated with poor prognosis of patients with colorectal cancer based on bioinformatics analysis (Wang et al., 2018; Huang et al., 2020), but it was not clear whether m6A modification involved in the regulation of colorectal progression. In consideration of its unfavorable role in colorectal cancer, LINC02257 is considered as a novel significant regulator in metastatic KIRC, particularly in m6A manner. There was no survival discrepancy between low expression and high expression of LINC01820 through KM survival analysis. We thought KM survival analysis on LINC01820 may be disturbed by other confounding factors, leading to LINC01820 was not related with prognosis, and multivariate Cox regression analysis could solve the problem. The result from multivariate Cox regression analysis showed that LINC01820 was an independent factor associated with prognosis. Based on the result, we preferred to believe LINC01820 was a prognosis-associated m6A-lncRNA. Currently, there were no articles reporting that LINC01820 is correlated with prognosis in other tumors. ROC curve illustrated a moderate predictive signature for the m6AlRsPI. The m6AlRsPI developed by the training cohort was tested by self-validation and the prognostic signature of LINC0257 was validated by external GEO cohort. These results supported m6AlRsPI was a robust and reasonable model for predicting prognosis. The analysis of biological signatures for m6AlRsPI based on two hub m6A-lncRNAs showed that the high m6AlRsPI group had more samples with gene mutation, more likely to cause epithelial–mesenchymal transition, and led to worse prognosis. As LINC01820 and LINC02257 did not regulate tumor behavior by lncRNA-miRNA-mRNA manner, RNA-binding proteins which connected with the two hub m6A-lncRNAs were investigated. Then, we found that modulated by the two hub m6A-lncRNAs, the mRNA surveillance signaling pathway, termed nonsense-mediated mRNA decay (NMD), has been found to be associated with mutation in human cancer by NMD-triggering manner (Lindeboom et al., 2016). This signaling pathway may play an important role in tumorigenesis and development of KIRC. The over-expression of the two hub m6A-lncRNAs in KIRC cell lines validated by qRT-PCR further indicated that they may influence tumor behavior, contributing to poor prognosis. These findings demonstrated that the two hub m6A-lncRNAs and m6AlRsPI were crucial for patients with metastatic KIRC to predict the prognosis and investigate the complex metastatic regulatory mechanism. Accordingly, they were significantly promising targets for treatment of patients with metastatic KIRC.

However, some limitations in our study should be taken into consideration. First, WGCNA was only performed on differentially expressed m6A-lncRNAs, other lncRNAs were excluded, causing some m6A-lncRNAs loss. Second, the red and turquoise modules had a weak correlation with the M stage. Third, the prognostic values of LINC01820 and m6AlRsPI cannot be validated by the external cohort currently. Fourth, as the database on m6A gene is updating, the screening of m6A-lncRNAs in our study was based on the current m6A dataset. Fifth, downstream molecular mechanisms for the two hub m6A-lncRNAs are unknown. It is required to conduct relevant experiments on the two hub m6A-lncRNAs to elucidate regulatory signaling pathways for metastatic KIRC.

## Conclusion

In the present study, hub m6A-lncRNAs in KIRC were screened based on WGCNA, the m6A-lncRNAs prognostic index (m6AlRsPI) was constructed and validated according to two hub m6A-lncRNAs linked with prognosis, and the biological signatures and prognostic values were investigated. The two hub m6A-lncRNAs can be looked upon as critical regulatory molecules in metastatic KIRC and as potential therapeutic targets.

## Data Availability

The datasets presented in this study can be found in online repositories. The names of the repository/repositories and accession number(s) can be found in the article/Supplementary Material.

## Abbreviations

m6A, N6-methyladenosine; KIRC, kidney renal clear cell carcinoma; DElncRNAs, differentially expressed lncRNAs; WGCNA, weighted gene co-expression network analysis; m6AlRsPI, m6A-lncRNAs prognostic index; ceRNA network, lncRNA-miRNA-mRNA network; GO, Gene Ontology; KEGG, Kyoto Encyclopedia of Genes and Genomes; EMT, epithelial-mesenchymal transition; ROC, receiver operating characteristic

## References

[B1] Bach D.-H. Lee S. K. (2018). Long Noncoding RNAs in Cancer Cells. Cancer Lett. 419, 152–166. 10.1016/j.canlet.2018.01.053 29414303

[B2] Camacho C. V. Choudhari R. Gadad S. S. (2018). Long Noncoding RNAs and Cancer, an Overview. Steroids 133, 93–95. 10.1016/j.steroids.2017.12.012 29317255

[B3] Chen X.-Y. Zhang J. Zhu J.-S. (2019). The Role of m6A RNA Methylation in Human Cancer. Mol. Cancer 18 (1), 103. 10.1186/s12943-019-1033-z 31142332 PMC6540575

[B4] Gutschner T. Diederichs S. (2012). The Hallmarks of Cancer. RNA Biol. 9 (6), 703–719. 10.4161/rna.20481 22664915 PMC3495743

[B5] Hsieh J. J. Purdue M. P. Signoretti S. Swanton C. Albiges L. Schmidinger M. (2017). Renal Cell Carcinoma. Nat. Rev. Dis. Primers 3, 17009. 10.1038/nrdp.2017.9 28276433 PMC5936048

[B6] Hua W. Zhao Y. Jin X. Yu D. He J. Xie D. (2018). METTL3 Promotes Ovarian Carcinoma Growth and Invasion through the Regulation of AXL Translation and Epithelial to Mesenchymal Transition. Gynecol. Oncol. 151 (2), 356–365. 10.1016/j.ygyno.2018.09.015 30249526

[B7] Huang X. Cai W. Yuan W. Peng S. (2020). Identification of Key lncRNAs as Prognostic Prediction Models for Colorectal Cancer Based on LASSO. Int. J. Clin. Exp. Pathol. 13 (4), 675–684. 32355515 PMC7191144

[B8] Kim J. Piao H.-L. Kim B.-J. Yao F. Han Z. Wang Y. (2018). Long Noncoding RNA MALAT1 Suppresses Breast Cancer Metastasis. Nat. Genet. 50 (12), 1705–1715. 10.1038/s41588-018-0252-3 30349115 PMC6265076

[B9] Langfelder P. Horvath S. (2008). WGCNA: an R Package for Weighted Correlation Network Analysis. BMC bioinformatics 9, 559. 10.1186/1471-2105-9-559 19114008 PMC2631488

[B10] Li J. Chen F. Peng Y. Lv Z. Lin X. Chen Z. (2020). N6-Methyladenosine Regulates the Expression and Secretion of TGFβ1 to Affect the Epithelial-Mesenchymal Transition of Cancer Cells. Cells 9 (2), 296. 10.3390/cells9020296 PMC7072279 31991845

[B11] Li T. Hu P.-S. Zuo Z. Lin J.-F. Li X. Wu fnm. (2019). METTL3 Facilitates Tumor Progression via an m6A-igf2bp2-dependent Mechanism in Colorectal Carcinoma. Mol. Cancer 18 (1), 112. 10.1186/s12943-019-1038-7 31230592 PMC6589893

[B12] Lin S. Choe J. Du P. Triboulet R. Gregory R. I. (2016). The M 6 A Methyltransferase METTL3 Promotes Translation in Human Cancer Cells. Mol. Cel. 62 (3), 335–345. 10.1016/j.molcel.2016.03.021 PMC4860043 27117702

[B13] Lindeboom R. G. H. Supek F. Lehner B. (2016). The Rules and Impact of Nonsense-Mediated mRNA Decay in Human Cancers. Nat. Genet. 48 (10), 1112–1118. 10.1038/ng.3664 27618451 PMC5045715

[B14] Ljungberg B. Campbell S. C. Cho H. Y. Jacqmin D. Lee J. E. Weikert S. (2011). The Epidemiology of Renal Cell Carcinoma. Eur. Urol. 60 (4), 615–621. 10.1016/j.eururo.2011.06.049 21741761

[B15] Luo X. Li H. Liang J. Zhao Q. Xie Y. Ren J. (2020). RMVar: an Updated Database of Functional Variants Involved in RNA Modifications. Nucleic Acids Res. 49, D1405–D1412. 10.1093/nar/gkaa811 PMC7779057 33021671

[B16] Meyer K. D. Jaffrey S. R. (2014). The Dynamic Epitranscriptome: N6-Methyladenosine and Gene Expression Control. Nat. Rev. Mol. Cel Biol 15 (5), 313–326. 10.1038/nrm3785 PMC4393108 24713629

[B17] Pan L. Li Y. Jin L. Li J. Xu A. (2020). TRPM2-AS Promotes Cancer Cell Proliferation through Control of TAF15. Int. J. Biochem. Cel Biol. 120, 105683. 10.1016/j.biocel.2019.105683 31887411

[B18] Peng P.-H. Chieh-Yu Lai J. Hsu K.-W. Wu K.-J. (2020). Hypoxia-induced lncRNA RP11-390F4.3 Promotes Epithelial-Mesenchymal Transition (EMT) and Metastasis through Upregulating EMT Regulators. Cancer Lett. 483, 35–45. 10.1016/j.canlet.2020.04.014 32353468

[B19] Qin Y. Liu X. Pan L. Zhou R. Zhang X. (2019). Long Noncoding RNA MIR155HG Facilitates Pancreatic Cancer Progression through Negative Regulation of miR‐802. J. Cel Biochem 120 (10), 17926–17934. 10.1002/jcb.29060 31161625

[B20] Rini B. I. Campbell S. C. Escudier B. (2009). Renal Cell Carcinoma. The Lancet 373 (9669), 1119–1132. 10.1016/S0140-6736(09)60229-4 19269025

[B21] Shang A. Wang W. Gu C. Chen C. Zeng B. Yang Y. (2019). Long Non-coding RNA HOTTIP Enhances IL-6 Expression to Potentiate Immune Escape of Ovarian Cancer Cells by Upregulating the Expression of PD-L1 in Neutrophils. J. Exp. Clin. Cancer Res. 38 (1), 411. 10.1186/s13046-019-1394-6 31533774 PMC6751824

[B22] Siegel R. L. Miller K. D. Jemal A. (2019). Cancer Statistics, 2019. CA A. Cancer J. Clin. 69 (1), 7–34. 10.3322/caac.21551 30620402

[B23] Tang B. Yang Y. Kang M. Wang Y. Wang Y. Bi Y. (2020). m6A Demethylase ALKBH5 Inhibits Pancreatic Cancer Tumorigenesis by Decreasing WIF-1 RNA Methylation and Mediating Wnt Signaling. Mol. Cancer 19 (1), 3. 10.1186/s12943-019-1128-6 31906946 PMC6943907

[B24] Thomas J. S. Kabbinavar F. (2015). Metastatic Clear Cell Renal Cell Carcinoma: A Review of Current Therapies and Novel Immunotherapies. Crit. Rev. oncology/hematology 96 (3), 527–533. 10.1016/j.critrevonc.2015.07.009 26299335

[B25] Wang H. Deng Q. Lv Z. Ling Y. Hou X. Chen Z. (2019). N6-methyladenosine Induced miR-143-3p Promotes the Brain Metastasis of Lung Cancer via Regulation of VASH1. Mol. Cancer 18 (1), 181. 10.1186/s12943-019-1108-x 31823788 PMC6902331

[B26] Wang Q. Chen C. Ding Q. Zhao Y. Wang Z. Chen J. (2020). METTL3-mediated m6A Modification of HDGF mRNA Promotes Gastric Cancer Progression and Has Prognostic Significance. Gut 69 (7), 1193–1205. 10.1136/gutjnl-2019-319639 31582403

[B27] Wang T. Kong S. Tao M. Ju S. (2020). The Potential Role of RNA N6-Methyladenosine in Cancer Progression. Mol. Cancer 19 (1), 88. 10.1186/s12943-020-01204-7 32398132 PMC7216508

[B28] Wang X. Zhou J. Xu M. Yan Y. Huang L. Kuang Y. (2018). A 15-lncRNA Signature Predicts Survival and Functions as a ceRNA in Patients with Colorectal Cancer. Cmar Vol. 10, 5799–5806. 10.2147/CMAR.S178732 PMC6248371 30510449

[B29] Wang Y. Lu J.-H. Wu Q.-N. Jin Y. Wang D.-S. Chen Y. X. (2019). LncRNA LINRIS Stabilizes IGF2BP2 and Promotes the Aerobic Glycolysis in Colorectal Cancer. Mol. Cancer 18 (1), 174. 10.1186/s12943-019-1105-0 31791342 PMC6886219

[B30] Wen S. Wei Y. Zen C. Xiong W. Niu Y. Zhao Y. (2020). Long Non-coding RNA NEAT1 Promotes Bone Metastasis of Prostate Cancer through N6-Methyladenosine. Mol. Cancer 19 (1), 171. 10.1186/s12943-020-01293-4 33308223 PMC7733260

[B31] Wu Y. Yang X. Chen Z. Tian L. Jiang G. Chen F. (2019). m6A-induced lncRNA RP11 Triggers the Dissemination of Colorectal Cancer Cells via Upregulation of Zeb1. Mol. Cancer 18 (1), 87. 10.1186/s12943-019-1014-2 30979372 PMC6461827

[B32] Xie J.-W. Huang X.-B. Chen Q.-Y. Ma Y.-B. Zhao Y.-J. Liu L.-C. (2020). m6A Modification-Mediated BATF2 Acts as a Tumor Suppressor in Gastric Cancer through Inhibition of ERK Signaling. Mol. Cancer 19 (1), 114. 10.1186/s12943-020-01223-4 32650804 PMC7350710

[B33] Yan J. Huang X. Zhang X. Chen Z. Ye C. Xiang W. (2020). LncRNA LINC00470 Promotes the Degradation of PTEN mRNA to Facilitate Malignant Behavior in Gastric Cancer Cells. Biochem. biophysical Res. Commun. 521 (4), 887–893. 10.1016/j.bbrc.2019.11.016 31711642

[B34] Yang Z. Ma J. Han S. Li X. Guo H. Liu D. (2020). ZFAS1 Exerts an Oncogenic Role via Suppressing miR-647 in an m6A-dependent Manner in Cervical Cancer. Ott Vol. 13, 11795–11806. 10.2147/OTT.S274492 PMC7680607 33235466

[B35] Yi Y.-C. Chen X.-Y. Zhang J. Zhu J.-S. (2020). Novel Insights into the Interplay between m6A Modification and Noncoding RNAs in Cancer. Mol. Cancer 19 (1), 121. 10.1186/s12943-020-01233-2 32767982 PMC7412851

[B36] Zhang C. Huang S. Zhuang H. Ruan S. Zhou Z. Huang K. (2020). YTHDF2 Promotes the Liver Cancer Stem Cell Phenotype and Cancer Metastasis by Regulating OCT4 Expression via m6A RNA Methylation. Oncogene 39 (23), 4507–4518. 10.1038/s41388-020-1303-7 32366907

[B37] Zhao B. S. Roundtree I. A. He C. (2017). Post-transcriptional Gene Regulation by mRNA Modifications. Nat. Rev. Mol. Cel Biol 18 (1), 31–42. 10.1038/nrm.2016.132 PMC5167638 27808276

[B38] Zheng Z.-G. Xu H. Suo S.-S. Xu X.-L. Ni M.-W. Gu L.-H. (2016). The Essential Role of H19 Contributing to Cisplatin Resistance by Regulating Glutathione Metabolism in High-Grade Serous Ovarian Cancer. Sci. Rep. 6, 26093. 10.1038/srep26093 27193186 PMC4872133

[B39] Zheng Z.-Q. Li Z.-X. Zhou G.-Q. Lin L. Zhang L.-L. Lv J.-W. (2019). Long Noncoding RNA FAM225A Promotes Nasopharyngeal Carcinoma Tumorigenesis and Metastasis by Acting as ceRNA to Sponge miR-590-3p/miR-1275 and Upregulate ITGB3. Cancer Res. 79 (18), 4612–4626. 10.1158/0008-5472.CAN-19-0799 31331909

