# Supplementary material for: Hub Long Noncoding RNAs with m6A Modification for Signatures and Prognostic Values in Kidney Renal Clear Cell Carcinoma
Source: Front Mol Biosci. 2021 Jul 6;8:682471. doi: 10.3389/fmolb.2021.682471 (PMC8290079; doi:10.3389/fmolb.2021.682471)
Supplement: Supplementary file 1 [file DataSheet3.DOCX]

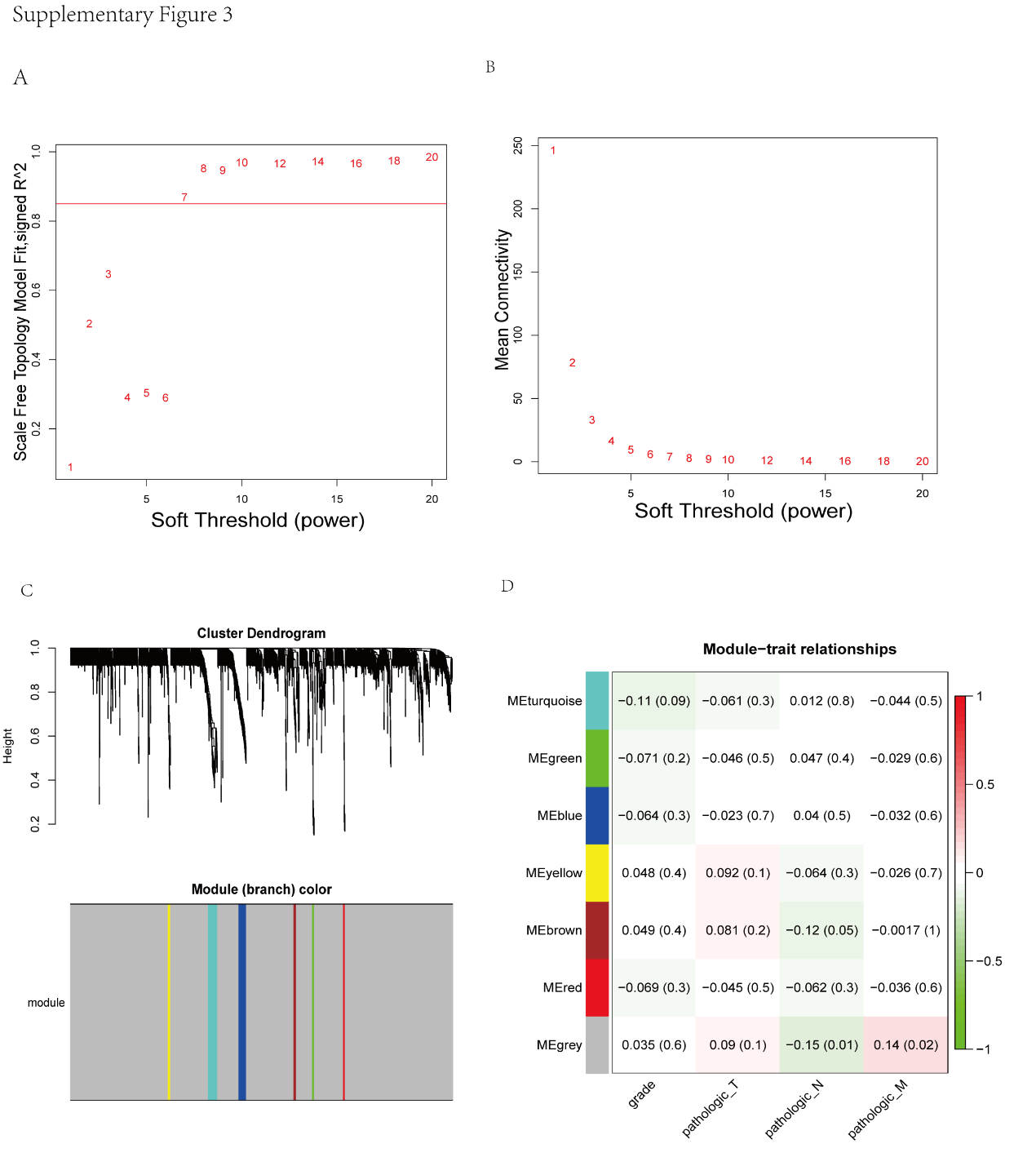


Supplementary Figure 3: WGCNA for production of gene modules in the testing cohort. (A) Analysis of the scale-free fit index for various soft threshold. And the soft threshold was set as 14. (B) Analysis of the mean connectivity for various soft threshold. (C) Dendrogram showing the clustered m6A-DElncRNAs. (D) Heatmap illustrating the association of the MEs with clinical characteristics (including grade and TNM staging). The module associated with M stage with P < 0.05 was selected.
