# Supplementary material for: Hub Long Noncoding RNAs with m6A Modification for Signatures and Prognostic Values in Kidney Renal Clear Cell Carcinoma
Source: Front Mol Biosci. 2021 Jul 6;8:682471. doi: 10.3389/fmolb.2021.682471 (PMC8290079; doi:10.3389/fmolb.2021.682471)
Supplement: Supplementary file 2 [file Table1.doc]

Supplementary Table 1: The clinical traits of TCGA training cohort and testing cohort

50% TCGA 50% TCGA TCGA parental

Traits cohort (training cohort) cohort (testing cohort) cohort

N = 272 N = 271 N = 543

Age

>=50/<50 211/61 223/48 434/109

Gender

male/female 181/91 171/100 352/191

Stage

i/ii/iii/iv 135/27/63/47 138/31/63/36 273/58/126/83

Grade

G1/G2/G3/G4 6/115/103/39 8/119/106/38 14/234/209/77

pathologic_T

T1/T2/T3/T4 145/37/85/5 134/33/98/6 279/70/183/11

pathologic_N

N0/N1 123/7 118/10 241/17

pathologic_M

M0/M1 220/43 211/36 431/79
