# Supplementary material for: Hub Long Noncoding RNAs with m6A Modification for Signatures and Prognostic Values in Kidney Renal Clear Cell Carcinoma
Source: Front Mol Biosci. 2021 Jul 6;8:682471. doi: 10.3389/fmolb.2021.682471 (PMC8290079; doi:10.3389/fmolb.2021.682471)
Supplement: Supplementary file 3 [file Table2.docx]

Supplementary Table 2: The value of Maximal Clique Centrality (MCC)

id MCC value

AC005291.1 12

AC005291.2 8

AC004817.3 8

FGF14-AS1 7

AP005131.1 7

PICSAR 6

MIR663AHG 4

AC089983.1 4

LINC00706 3

ATP11A-AS1 3

AC037487.2 3

LINC02257 2

AL583824.1 2

LINC01820 2

LY86-AS1 2

AL031123.1 2

ITPK1-AS1 2

FOXN3-AS2 2

AC087257.1 2

AP005131.4 2

PRRT3-AS1 2

LINC01655 1

LINC01505 1

LINC01290 1

CERS3-AS1 1

AL627309.5 1

AL355870.1 1

AL157896.1 1

AC234582.2 1

AC104984.3 1

LINC01632 1

AC078980.1 1

AL390061.1 1

AC046143.1 1

AC138305.1 1

AC040174.1 1

TBL1XR1-AS1 1

SHANK2-AS1 1

AC012404.1 1

AL355516.1 1

AC104002.3 0

AC092115.2 0

ELMO1-AS1 0

PROX1-AS1 0

AC015819.2 0

AC011595.1 0

AC000068.3 0
