# Supplementary material for: Hub Long Noncoding RNAs with m6A Modification for Signatures and Prognostic Values in Kidney Renal Clear Cell Carcinoma
Source: Front Mol Biosci. 2021 Jul 6;8:682471. doi: 10.3389/fmolb.2021.682471 (PMC8290079; doi:10.3389/fmolb.2021.682471)
Supplement: Supplementary file 4 [file DataSheet2.docx]

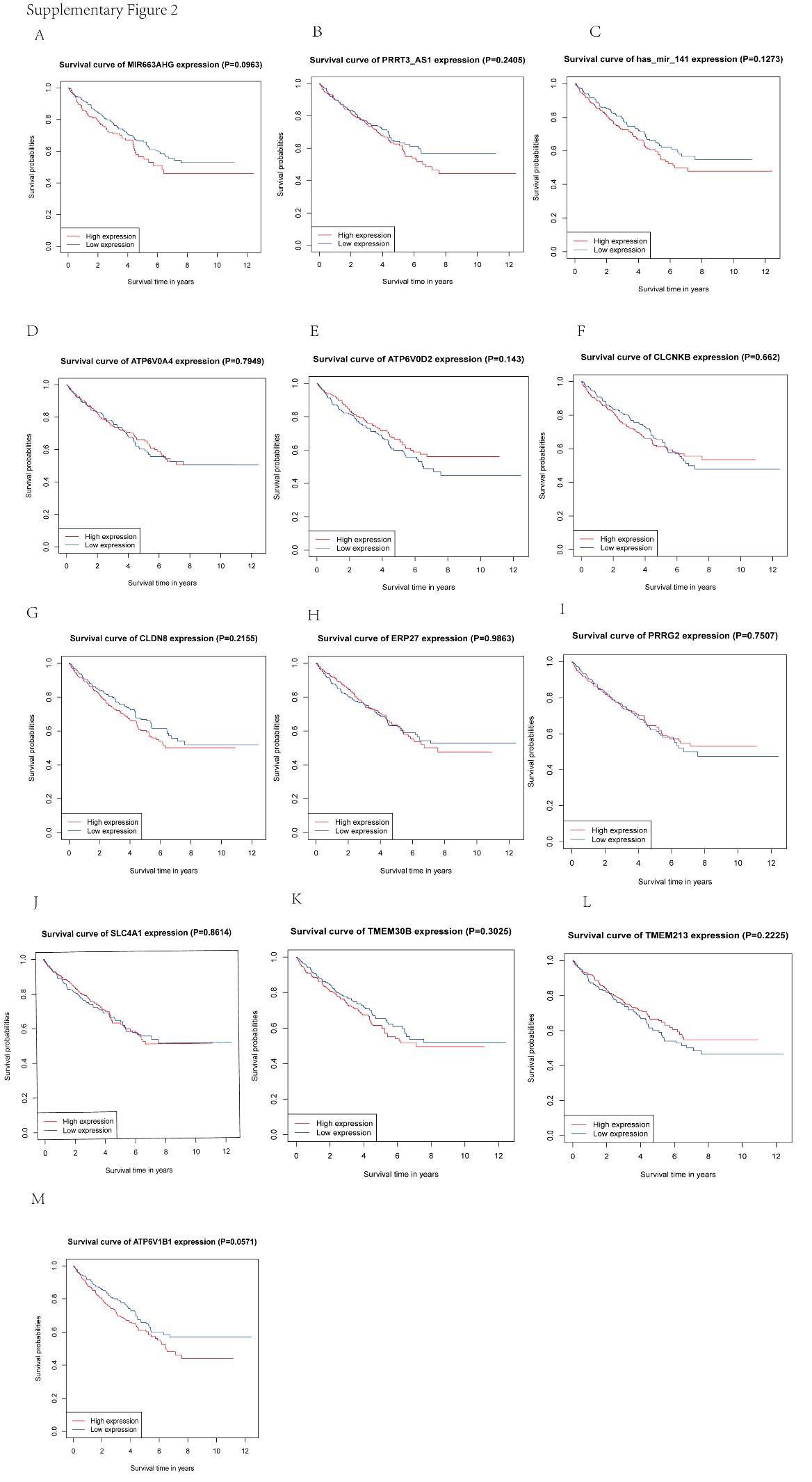


Supplementary Figure 2: Survival analysis for molecules within ceRNA network. (A-B) Survival analysis for lncRNAs within ceRNA network. (C) Survival analysis for miRNA. (D-M) Survival analysis for mRNAs with no statistical significance.
