# Supplementary material for: Hub Long Noncoding RNAs with m6A Modification for Signatures and Prognostic Values in Kidney Renal Clear Cell Carcinoma
Source: Front Mol Biosci. 2021 Jul 6;8:682471. doi: 10.3389/fmolb.2021.682471 (PMC8290079; doi:10.3389/fmolb.2021.682471)
Supplement: Supplementary file 5 [file DataSheet1.DOCX]

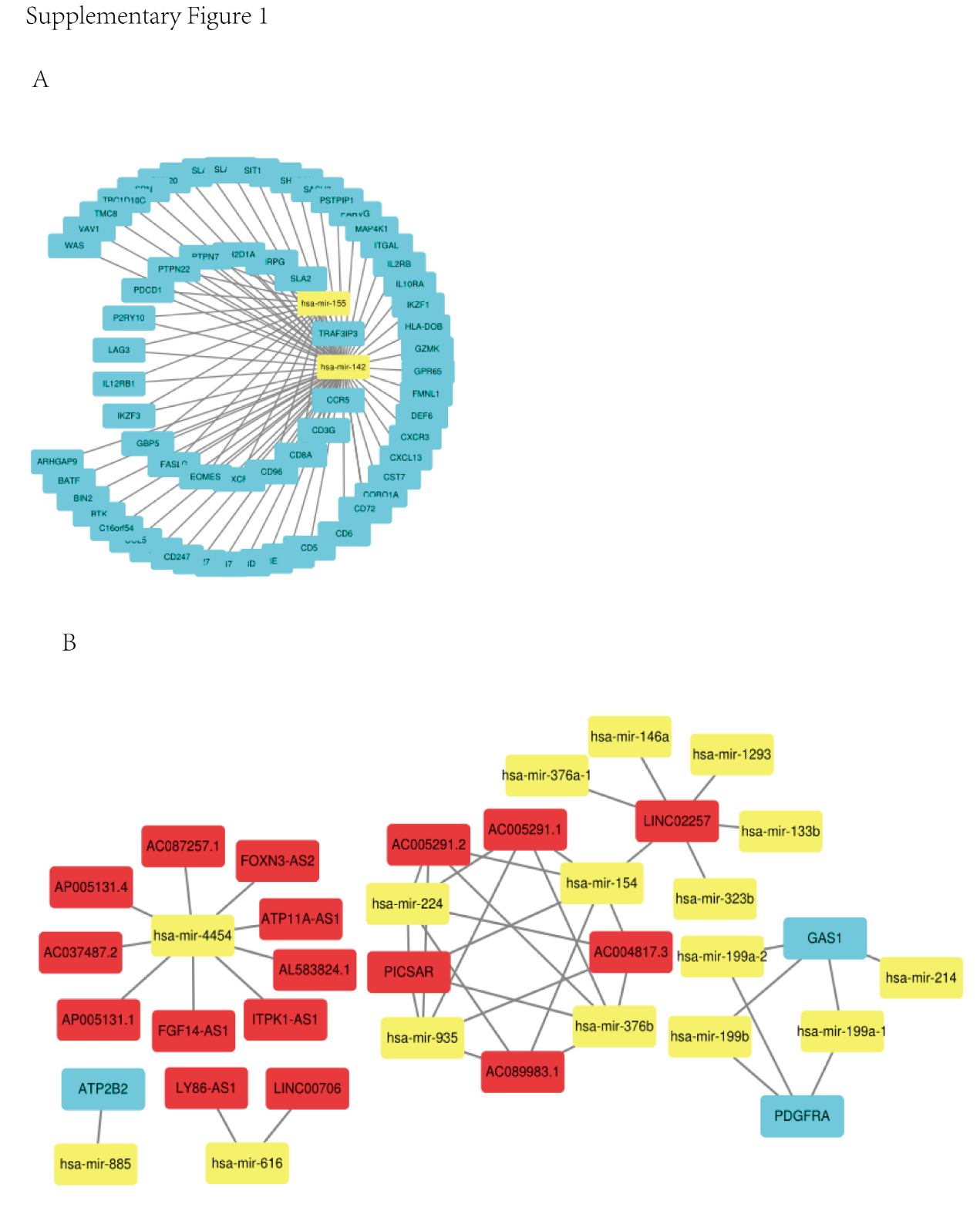


Supplementary Figure 1: Correlation networks. (A) lncRNA-miRNA correlation networks. (B) miRNA-mRNA correlation networks.
